# Mice Lacking Cerebellar Cortex and Related Structures Show a Decrease in Slow-Wave Activity With Normal Non-REM Sleep Amount and Sleep Homeostasis

**DOI:** 10.3389/fnbeh.2022.910461

**Published:** 2022-06-02

**Authors:** Tomoyuki Fujiyama, Henri Takenaka, Fuyuki Asano, Kazuya Miyanishi, Noriko Hotta-Hirashima, Yukiko Ishikawa, Satomi Kanno, Patricia Seoane-Collazo, Hideki Miwa, Mikio Hoshino, Masashi Yanagisawa, Hiromasa Funato

**Affiliations:** ^1^International Institute for Integrative Sleep Medicine (WPI-IIIS), University of Tsukuba, Tsukuba, Japan; ^2^Department of Biochemistry and Cellular Biology, National Institute of Neuroscience, National Center of Neurology and Psychiatry (NCNP), Kodaira, Japan; ^3^Department of Neuropsychopharmacology, National Institute of Mental Health, National Center of Neurology and Psychiatry (NCNP), Kodaira, Japan; ^4^Life Science Center for Survival Dynamics, Tsukuba Advanced Research Alliance, University of Tsukuba, Tsukuba, Japan; ^5^Department of Molecular Genetics, University of Texas Southwestern Medical Center, Dallas, TX, United States; ^6^Department of Anatomy, Graduate School of Medicine, Toho University, Tokyo, Japan

**Keywords:** cerebellum, sleep, delta power, fear conditioning, *Ptf1a*, sleep spindle

## Abstract

In addition to the well-known motor control, the cerebellum has recently been implicated in memory, cognition, addiction, and social behavior. Given that the cerebellum contains more neurons than the cerebral cortex and has tight connections to the thalamus and brainstem nuclei, it is possible that the cerebellum also regulates sleep/wakefulness. However, the role of the cerebellum in sleep was unclear, since cerebellar lesion studies inevitably involved massive inflammation in the adjacent brainstem, and sleep changes in lesion studies were not consistent with each other. Here, we examine the role of the cerebellum in sleep and wakefulness using mesencephalon- and rhombomere 1-specific *Ptf1a* conditional knockout (*Ptf1a* cKO) mice, which lack the cerebellar cortex and its related structures, and exhibit ataxic gait. *Ptf1a* cKO mice had similar wake and non-rapid eye movement sleep (NREMS) time as control mice and showed reduced slow wave activity during wakefulness, NREMS and REMS. *Ptf1a* cKO mice showed a decrease in REMS time during the light phase and had increased NREMS delta power in response to 6 h of sleep deprivation, as did control mice. *Ptf1a* cKO mice also had similar numbers of sleep spindles and fear memories as control mice. Thus, the cerebellum does not appear to play a major role in sleep-wake control, but may be involved in the generation of slow waves.

## Introduction

The cerebellum has been well characterized for its role in motor coordination and learning, but recently the cerebellum has been recognized to be involved in other functions, such as memory (Sacchetti et al., 2004; Strata, 2015; Frontera et al., 2020), cognition (Sokolov et al., 2017; Schmahmann, 2019), and social behavior (Carta et al., 2019; Van Overwalle et al., 2020). Consistently, cerebellar lesions and abnormalities are linked to several psychiatric disorders, including autism (Wang et al., 2014; Van Overwalle et al., 2020) and schizophrenia (Andreasen and Pierson, 2008; Moberget and Ivry, 2019).

The findings that individuals with cerebellar ischemia or degeneration often suffer from daytime sleepiness, insomnia and sleep-related symptoms (Pedroso et al., 2011; DelRosso and Hoque, 2014; Song and Zhu, 2021) suggest the role of the cerebellum in sleep/wake regulation. Electrolytic lesions of the fastigial nucleus in cats led to drowsiness (Giannazzo et al., 1968). Similarly, surgical removal of the entire cerebellum in cats resulted in an increase in drowsiness and rapid eye movement sleep (REMS) and a decrease in wakefulness and non-rapid eye movement sleep (NREMS) (Cunchillos and De Andrés, 1982). Reflecting that sleep-wake involves changes in the overall brain state, neural activity in the cerebellum also changes in response to the sleep-wake state (Canto et al., 2017), but studies of human cerebellar diseases and lesions in cats suggest that the cerebellum can be more actively involved in sleep-wake regulation. Consistently, multiple-unit recordings of Purkinje cells and deep cerebellar nuclei neurons in mice showed increased or decreased firing activities, respectively, prior to the transition from NREMS to wakefulness (Zhang et al., 2020). The cerebellum contains more neurons than the cerebral cortex (Herculano-Houzel, 2009) and has tight connections to the thalamus and brain stem (Gallay et al., 2008). However, surgical removal of the cerebellum inevitably leads to extensive inflammation and gliosis in those areas in the brainstem that are adjacent to the cerebellum and involved in wake maintenance and REMS regulation (Scammell et al., 2017; Liu and Dan, 2019), and the cerebellum is too large to be a target site for optogenetic or pharmacogenetic manipulation. Therefore, the role of the cerebellar system in sleep/wake behavior and in neural oscillations remains unclear.

Pancreas transcription factor 1a (*Ptf1a*) is a basic helix-loop-helix (bHLH) transcription factor that plays important roles in the development of the central nervous system and pancreas (Kawaguchi et al., 2002; Hoshino et al., 2005). *Ptf1a* is required for the development of the cerebellar system, including all cerebellar GABAergic neurons (Hoshino et al., 2005; Hori and Hoshino, 2012) and climbing fiber neurons in the inferior olivary nucleus (Yamada et al., 2007; Fujiyama et al., 2009), and mice lacking a presumable rhombomere 1 (r1) enhancer region of *Ptf1a* have no apparent cerebellum and are called “*cerebelless*” (Hoshino et al., 2005). In addition to the cerebellum, *Ptf1a* is also expressed in the developing hypothalamus. *Nkx2.1-Cre; Ptf1a*^*flox*/*del*^ mice showed that hypothalamic *Ptf1a* is required for sexual differentiation of the brain and body (Fujiyama et al., 2018). Along this line, *En1*^*Cre*/+^*; Ptf1a*^*flox*/*flox*^ mice, which lack the cerebellar cortex and related structures, can be used to investigate the role of the cerebellum in sleep/wake regulation and in the generation of oscillations associated with vigilance states.

Here, we show that *En1*^*Cre*/+^*; Ptf1a*^*flox*/*flox*^ mice have lower EEG delta density in all vigilance states and decreased REMS amount. Although mice lack the cerebellar system, they have normal wake and NREMS amount and normal homeostatic responses to sleep deprivation.

## Materials and Methods

### Animals

*Ptf1a* floxed mice (*Ptf1a*^*flox*^), *Ptf1a*^*cre*^ mice (Fujiyama et al., 2018), *Z/AP* mice (Jackson Laboratory #003919) and *En1*^*Cre*^ mice (Jackson Laboratory #007916) were used in this study. All experimental protocols were approved (Protocol #180094) and conducted following the guidelines established by the Institutional Animal Care and Use Committee of the University of Tsukuba. Mice were housed under a 12:12-h light/dark cycle and controlled temperature and humidity conditions with light onset at zeitgeber time 0 (ZT0). Food and water were available *ad libitum*. Mice were weaned at 4 weeks of age and were housed in groups of four or five. All mice were maintained on a C57BL/6 background.

### EEG/EMG Electrode Implantation Surgery

At 3–4 months of age, male mice were implanted with electroencephalography/electromygography (EEG/EMG) electrodes with four EEG electrode pins and 2 flexible stainless EMG wires under anesthesia using isoflurane (4% for induction, 2.5% for maintenance) as described previously (Miyoshi et al., 2019). An EEG/EMG electrode insulator was attached to the skull using dental cement. The EEG electrode pins were placed on the frontal and occipital cortices [anteroposterior (AP): 0.5 mm, mediolateral (ML): 1.3 mm, dorsoventral (DV): −1.3 mm and AP: −4.5 mm, ML: 1.3 mm, DV: −1.3 mm]. The EMG wires were inserted into the neck muscles. All mice were allowed at least 4–7 days for recovery from surgery. After the recovery period, all mice were attached to a tether cable and then allowed to habituate to recording condition for 7 days.

### EEG/EMG Recording and Analysis

EEG/EMG recordings were analyzed as described (Miyoshi et al., 2019). The recording room was kept under a 12:12 h light/dark cycle and a constant temperature (24–25°C). EEG/EMG signals were obtained using LabVIEW-based software (National Instruments) at a sampling rate of 250 Hz. EEG/EMG data were visualized and semiautomatically analyzed by MATLAB-based software (Math Works), followed by visual staging. The sleep/wake state in each 20-s epoch was classified into NREMS, REMS or wakefulness. Wakefulness was scored based on the presence of fast EEG, high amplitude and variable EMG. NREMS was staged based on high amplitude, delta (1–4 Hz) frequency EEG and low EMG tonus, whereas REMS was staged by theta (6–9 Hz)-dominant EEG oscillation and muscle atonia. For analysis of baseline sleep/wake behavior, EEG/EMG signals were recorded for 2 consecutive days from the onset of the light phase, ZT0. The total time spent in wakefulness, NREMS and REMS was derived by summing the total number of 20-s epochs in each state. Epochs that contained movement artifacts were included in the time-domain analysis but excluded from the spectral analysis. Almost all epochs were included in the spectrum analysis for NREMS and REMS. Mean episode duration was determined by dividing the total time spent in each state by the number of episodes of that state. EEG signals were subjected to fast Fourier transform (FFT) analysis from 1 to 30 Hz with a 1-Hz bin using MATLAB-based custom software. EEG power density in each frequency bin was expressed as a ratio of the sum of the higher frequency range (16–30 Hz) of all sleep/wake states. Hourly delta density during NREMS indicates hourly averages of delta density, which is the ratio of delta power (1–4 Hz) to the sum of 16–30-Hz bins EEG power at all NREMS epochs. For sleep deprivation, mice were sleep-deprived for 6 h from the onset of light phase ZT0 by gentle handling. During sleep deprivation, food and water were available. For evaluation of the effect of sleep deprivation, NREMS delta power during the first 2 h after sleep deprivation was expressed relative to the same ZT of the basal recording or relative to the mean of the basal recording.

### Sleep Spindle Analysis

Sleep spindles were detected using an automated algorithm using MATLAB script as previously described (Uygun et al., 2019). Briefly, EEG data were bandpass-filtered across the sigma frequency range (10–15 Hz, Butterworth Filter). Next, the root-mean-squared (RMS) power was calculated to provide an upper envelope of the bandpass signal. The RMS data were then exponentially transformed to accentuate spindle-generated signals above baseline. Putative spindle peaks were identified in transformed data by crossing of an upper-threshold value, set as 3.5 × the mean RMS EEG power across all states for each mouse. Additional detection criteria included a minimum duration of 0.5 s, based on crossing of a lower threshold set at 1.2 × mean RMS power, and a minimum inter-event interval of 0.5 s. This algorithm was used to score the total number of spindles and spindle density during NREMS (i.e., the number of NREMS spindles/NREMS min) across the 24 h NREMS state, the duration of each spindle during NREMS (seconds), the median peak of the greatest value of each spindle during NREMS, and the amplitude of the spindles during NREMS that were normalized to 24 h NREMS at ZT0–ZT4.

### Immunohistochemistry and Alkaline Phosphatase Staining

Under anesthesia with 4% isoflurane, adult male mice were transcardially perfused with phosphate-buffered saline (PBS) followed by 4% paraformaldehyde in PBS. The brains were post-fixed with 4% paraformaldehyde in PBS for 3–4 h at 4°C. Fixed brains were cryoprotected by overnight immersion in 15 and 30% sucrose in PBS, progressively at 4°C, embedded in Tissue-Tek O.C.T. compound (Sakura Finetek, Japan) and cryosectioned at 40 μm. The primary antibodies used in this study were anti-TBR1 (1:500; rabbit; EPR8138; Abcam) and anti-calbindin antibodies (1:500; rabbit; AB1778; Millipore). Immunohistochemistry was performed as described previously (Fujiyama et al., 2009). For alkaline phosphatase (AP) staining, sections were preincubated in PBS for 30 min at 75°C and then washed with Tris-HCl-buffered saline (TS) 9.5 (0.1 M Tris-HCl, pH 9.5, 0.1 M NaCl, and 10 mM MgCl_2_) at room temperature for 10 min. Coloring reactions were performed with nitro blue tetrazolium/5-bromo-4-chloro-3-indolyl phosphate (NBT/BCIP) (Roche) diluted 1:50 with TS9.5 for 30 min to several hours and then washed with 0.1% Tween and 2 mM MgCl_2_ in PBS. Samples were dehydrated and mounted with Entellan new (Merck). Fluorescence imaging was carried out using an LSM 780 confocal microscope system (Carl Zeiss). Images of Nissl-stained and AP-stained sections were acquired using NanoZoomer-XR system (Hamamatsu) and Leica application suite microscope system (Leica).

### CT Imaging

At 3 months of age, mice were sacrificed by CO_2_ exposure and computed tomography (CT) images of the skull were acquired using a 3D micro X-ray CT scanner R_mCT2 (Rigaku). CT images of two males from each genotype group (*Ptf1a*^*flox*/*flox*^ and *En1*^*Cre*/+^*; Ptf1a*^*flox*/*flox*^) were collected. Skull shape images were visualized using Metabolic Analysis software (Rigaku).

### Body Weight Measurement

Body weight was measured weekly from the age of 8 to 12 weeks. At 6 weeks of age, male mice were assigned to a chow diet (MF; Oriental Yeast). The normal chow diet provided 3.6 kcal/g (61% carbohydrate, 26% protein, and 13% fat).

### Glucose Tolerance Test

Glucose tolerance test (GTT) was performed as described (Seoane-Collazo et al., 2018). At 16–24 weeks of age, 50% glucose dextrose (FUJIFILM Wako) in 0.9% NaCl saline was intraperitoneally injected at once to 2.0 g glucose/kg body weight. Glucose levels were measured between ZT0 and ZT3 in blood from the tail vein using Glutest kits (Sanwa Kagaku).

### Footprint Test and Rotarod Test

At 16–20 weeks of age, footprint test, followed by rotarod test were performed between ZT1 and ZT8 by investigators blinded to the genotype (Miwa et al., 2021). For the footprint test, the forelimbs and hindlimbs of mice were painted with red or blue acrylic paint, respectively, and the mice were allowed to walk along a 45-cm-long 6-cm-wide runway with 10-cm-high walls. A fresh sheet of paper was placed on the floor of the runway for each run. Each mouse was tested twice. The footprint patterns of the fore and hind paws were evaluated in terms of the following parameter: stride length, which was measured as the average distance in forward movement between each stride. For the rotarod test, a mouse was placed on a rod (3 cm diameter; O'Hara & Co., Ltd., Tokyo, Japan) and was tested under the following conditions: the rod was accelerated (4–40 rpm over 3 min; three trials for 1 day at 2 h intervals). The amount of time that each mouse was able to maintain its balance on the rod was measured.

### Fear Conditioning

At 12–16 weeks of age, male mice were subjected to fear-conditioning experiments in an isolation chamber (Kumar et al., 2020). The conditioning chamber consisted of clear acrylic walls and a stainless-steel grid floor (width × depth × height, 310 × 240 × 210 mm; MED Associates, USA). The grid floor had bars (2.0-mm diameter) spaced 6.0 mm apart allowing the delivery of electric shocks. The conditioning chamber was placed inside an isolated behavioral chamber to keep the visual and sensory cues constant. A camera was placed at the top of the behavioral chamber and was remotely controlled so that mice could not see the experimenter. For scoring of freezing behavior, automated video-based freezing detection software was used (FreezeFrame5, Actimetrics). Freezing was defined as a ≥ 1 s continuous absence of movements except for breathing. For auditory fear conditioning, mice were placed in the conditioning context (context A) for 360 s in total, and three tones (30 s each, 2,800 Hz, 85 dB) were played at 120, 210, and 300 s, with each tone terminating with a 2-s foot shock (0.75 mA). Before each conditioning session, a white acrylic drop pan under the grid floor was cleaned with 70% ethanol water, which also provided a background odor. During the contextual memory test, mice were placed in context A for 300 s in total without auditory/electric stimuli or cleaning. During the cued memory test, mice were placed in a non-conditioned context (context B) for 360 s in total, and the tone was played during the last 180 s of the session. Context B was similar to the conditioning context except that the floor and sides of the chamber were covered with white plastic sheets, a piece of cardboard with a blue and white rectangular pattern was affixed to the back wall, and ethanol was not used for cleaning.

### Statistics

Sample sizes were determined using R software based on averages and standard deviations that were obtained from small-scale experiments or from our previous data. No method of randomization was used in any of the experiments. The experimenters were blinded to genotypes and treatment assignments. All statistical comparisons were performed using Prism 8 (GraphPad software). All data are presented as the mean ± standard error of the mean (SEM). Details of each experiment are included in the figure legends (animal number, genotype, and statistical test). *P*-value < 0.05 was considered statistically significant.

## Results

### Generation of *Ptf1a* Conditional Knockout Mice That Lack Cerebellar Cortex

**Figure 1:**
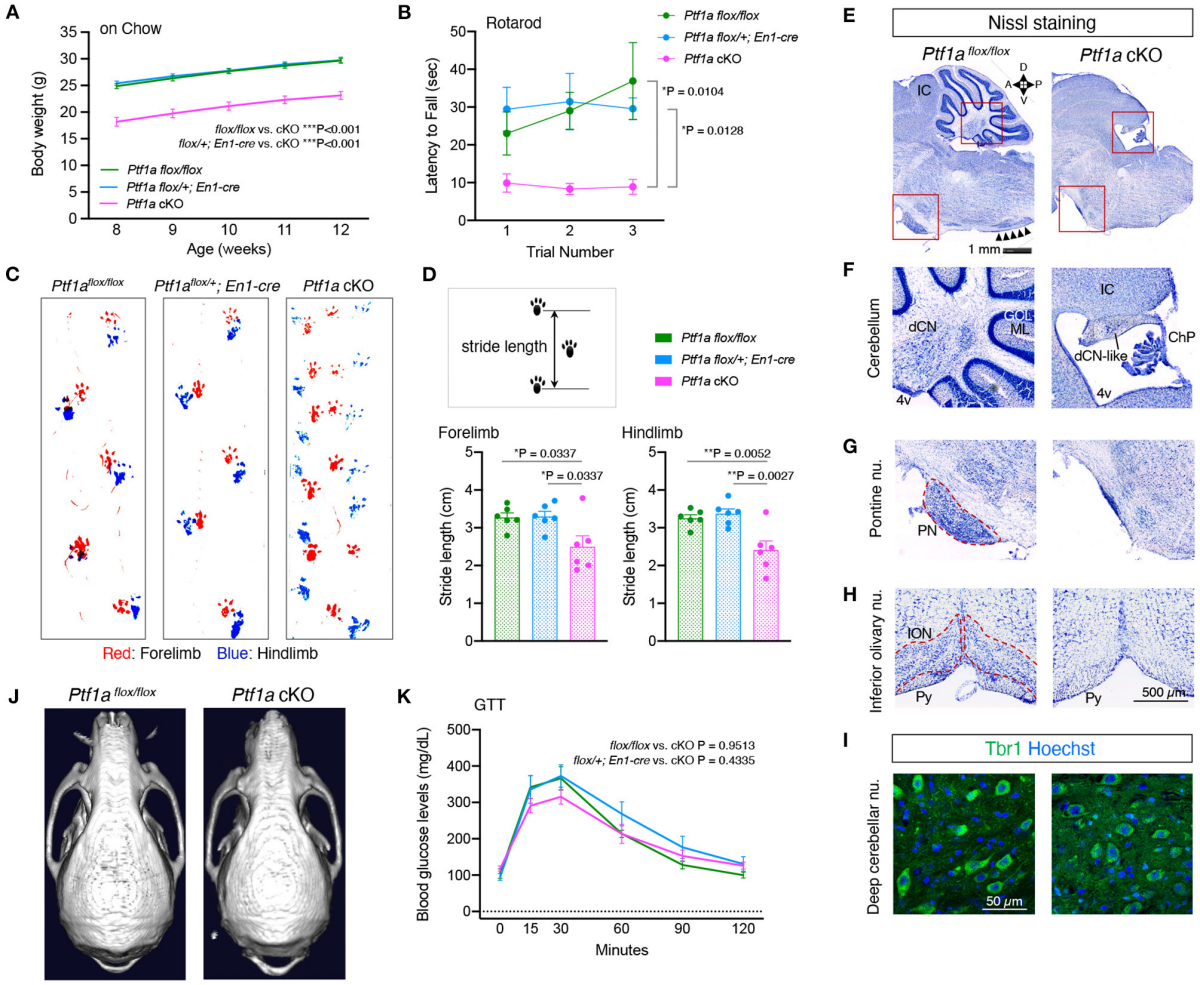
Loss of *Ptf1a* in the *En1*-lineage results in lack of cerebellar cortex. **(A)** Body weight curves of male *Ptf1a* cKO and two control groups (*Ptf1a*^*flox*/*flox*^, or *En1*^*cre*/+^*; Ptf1a*^*flox*/+^) at the young-adult stages (*n* = 15 for *Ptf1a*^*flox*/*flox*^, *n* = 25 for *En1*^*cre*/+^*; Ptf1a*^*flox*/+^, *n* = 16 for *En1*^*cre*/+^*; Ptf1a*^*flox*/*flox*^, two-way ANOVA with Sidak's multiple comparisons test). **(B)** Latency to fall on the rotarod over three training trials in adult male *Ptf1a* cKO and control groups (*n* = 11 for *Ptf1a*^*flox*/*flox*^, *n* = 9 for *En1*^*cre*/+^*; Ptf1a*^*flox*/+^, *n* = 10 for *En1*^*cre*/+^*; Ptf1a*^*flox*/*flox*^, two-way ANOVA with Sidak's multiple comparisons test). **(C)** Representative walking footprint patterns of each genotype. An uncoordinated pattern is observed in *Ptf1a* cKO mice. **(D)** Stride length is measured in the gait analysis. Quantitative analyses of forelimb (left panel) and hindlimb (right panel) footprints (*n* = 6 for each genotype, one-way ANOVA with Holm-Sidak multiple comparisons test). **(E–H)** Nissl staining of midline sagittal sections **(E–G)** and coronal sections **(H)** from adult *Ptf1a* cKO (right) and control (left) brains showing lack of cerebellar cortex, pontine nuclei, and inferior olivary nucleus. Arrowheads in **(E)** delineate the laminar structure at the ION region observed in control, but not in cKO mutant. IC, inferior colliculus; dCN, deep cerebellar nucleus; GCL, granule cell layer; ML, molecular layer; 4v, fourth ventricle; dCN-like, residual deep cerebellar nucleus; ChP, choroid plexus; PN, pontine nuclei; ION, inferior olivary nucleus; Py, pyramidal tract. **(I)** Immunofluorescence of cerebellar sections for the dCN glutamatergic projection neuron marker Tbr1 in an adult *Ptf1a* cKO compared to a control. **(J)** Representative skull CT images of adult *Ptf1a* cKO and control mice. **(K)** Glucose tolerance test in 4 to 6-month-old male *Ptf1a* cKO and control groups (*n* = 7 for *Ptf1a*^*flox*/*flox*^, *n* = 6 for *En1*^*cre*/+^*; Ptf1a*^*flox*/+^, *n* = 7 for *En1*^*cre*/+^*; Ptf1a*^*flox*/*flox*^, one-way ANOVA with Tukey's multiple comparisons test). All data are expressed as the mean ± S.E.M.

To generate mice lacking cerebellar structures, *Ptf1a* was deleted in the developing mesencephalon and rhombomere 1 hindbrain region using *En1*^*Cre*^ mice (Kimmel et al., 2000). *En1*^*Cre*/+^*; Ptf1a*^*flox*/*flox*^ mice, are hereafter referred to as *Ptf1a* cKO mice. Adult *Ptf1a* cKO mice appeared healthy but small and showed uncoordinated movements. The body weights of male *Ptf1a* cKO mice were significantly lower than those of male control animals (*Ptf1a*^*flox*/*flox*^, or *En1*^*cre*/+^*; Ptf1a*^*flox*/+^) at the age of 8–12 weeks (Figure 1A). To evaluate motor coordination, the accelerating rotarod and gait patterning tests were conducted. In the rotarod test, the latency to fall in male *Ptf1a* cKO mice was significantly shorter than those in male control groups (Figure 1B). Compared to the male control group, male *Ptf1a* cKO mice showed uncoordinated and shortened forelimb and hindlimb movement (Figures 1C,D; Supplementary Video 1), as well as tremor and ataxic gait phenotypes in the *cerebelless* mutant mice (*Ptf1a*^*cbll*/*cbll*^) (Hoshino et al., 2005).

Nissl staining of brain sections showed that *Ptf1a* cKO mice lacked the entire cerebellum except rudimental deep cerebellar nucleus (Figures 1E,F). Neurons in the deep cerebellar nucleus-like structure were immunopositive for TBR1 (Figure 1I), which is expressed in a subset of glutamatergic neurons in the fastigial nucleus (Fink et al., 2006), but immunonegative for calbindin antibody (data not shown), a marker for Purkinje cells. In addition to the cerebellum, the pontine nuclei and the inferior olivary nucleus, which are known as the precerebellar systems that send mossy fiber or climbing fiber projections to the cerebellar cortex, respectively, were also missing in adult *Ptf1a* cKO brains (Figures 1G,H). There were no apparent differences in the cerebral cortex, hippocampus, or thalamus (data not shown). These morphological features observed in *Ptf1a* cKO mice were basically identical to those in *cerebelless* mutant mice (Hoshino et al., 2005). The occipital bone of *Ptf1a* cKO mice seemed to be shrunken, which may be due to lack of the cerebellum, but the frontal and parietal bones and cortices of *Ptf1a* cKO mice were apparently normal (Figure 1J; Supplementary Figures 1A,B), where EEG electrode pins were placed.

Since *Ptf1a* is required for the development of the pancreas (Kawaguchi et al., 2002), we performed GTT. Adult male *Ptf1a* cKO mice exhibited blood glucose levels similar to those of male controls (Figure 1K). These results indicate that *Ptf1a* cKO mice lack the cerebellar cortex and related precerebellar structures but have normal pancreatic function.

### *Ptf1a* cKO Mice Showed Decreased Slow-Wave Activity During the Sleep-Wake Cycle

**Figure 2:**
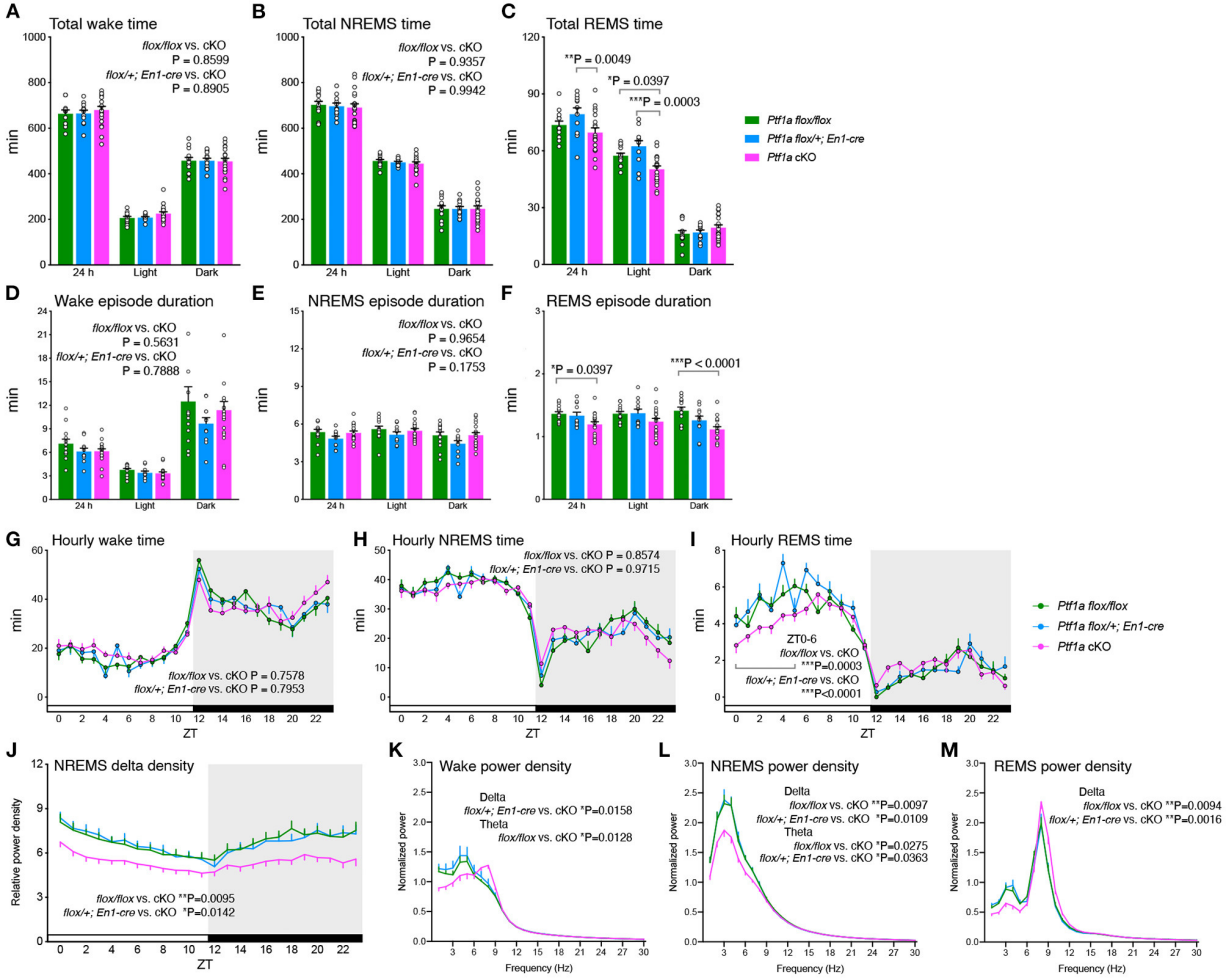
Sleep/wake behavior of adult male *Ptf1a* cKO mice. **(A–C)** Time spent in wake **(A)**, NREMS **(B)**, and REMS **(C)** of *Ptf1a* cKO and control mice over 24 h and during the light and dark phases. Two-way ANOVA with Sidak's multiple comparisons test. **(D–F)** Episode duration of wake **(D)**, NREMS **(E)**, and REMS **(F)** of *Ptf1a* cKO and control mice. Two-way ANOVA with Sidak's multiple comparisons test. **(G–I)** Daily variations in wake **(G)**, NREMS **(H)**, and REMS **(I)**. Mixed-effects analysis followed by Tukey's test for the hourly time in early light phases (ZT0-6). **(J)** Hourly NREMS delta power density over 24 h. Mixed-effects analysis followed by Sidak's test. **(K–M)** EEG power spectra of *Ptf1a* cKO and control mice during wake **(K)**, NREMS **(L)**, and REMS **(M)**. Mixed-effects analysis followed by Sidak's test for the delta- and theta-ranges. *n* = 13 for *Ptf1a*^*flox*/*flox*^, *n* = 11 for *En1*^*cre*/+^*; Ptf1a*^*flox*/+^, *n* = 19 for *En1*^*cre*/+^*; Ptf1a*^*flox*/*flox*^. Data are the mean ± S.E.M. *P < 0.05. **P < 0.01. ***P < 0.001.

Next, we performed EEG/EMG recording to investigate sleep/wakefulness in *Ptf1a* cKO mice. *Ptf1a* cKO mice exhibited total time spent in both wake and NREMS states similar to the two control groups (Figures 2A,B). *Ptf1a* cKO mice showed a reduction in total REMS time during the 24 h and light phases compared to *En1*^*Cre*/+^*; Ptf1a*^*flox*/+^ mice (Figure 2C). For episode duration, there were no significant differences in wake and NREMS episode durations (Figures 2D,E), whereas *Ptf1a* cKO mice showed a shorter REMS episode duration mainly during the dark phase than *Ptf1a*^*flox*/*flox*^ mice (Figure 2F). There was no significant difference in the daily total time spent or episode duration in any vigilance state between the two control genotypes. There were no significant differences in wake, NREMS, and REMS episode numbers among the genotypic groups (Supplementary Figures 2A–C). The diurnal variations of each state showed a sustained tendency toward shorter REMS time during the anterior half of the light phase in *Ptf1a* cKO mice (Figures 2G–I). EEG spectral analysis revealed that the hourly NREMS delta density of *Ptf1a* cKO mice was lower than that of the two control groups (Figure 2J). During wakefulness, *Ptf1a* cKO mice had a lower delta range (1–4 Hz) power than *En1*^*Cre*/+^*; Ptf1a*^*flox*/+^ mice and had a higher theta range (6–9 Hz) power than *Ptf1a*^*flox*/*flox*^ mice (Figure 2K). During NREMS, *Ptf1a* cKO mice had lower power in both the delta and theta ranges than the two control groups (Figure 2L). During REMS, *Ptf1a* cKO mice had a lower theta range power than the control groups (Figure 2M). As previously reported, a small number of r1-derived *Ptf1a*-linaege cells exit in the pons (Yamada et al., 2007). However, *Ptf1a*-lineage cells were not recognized in the pedunculopontine and laterodorsal tegmental nuclei (PPT/LDTg), which are involved in the regulation of REMS (Scammell et al., 2017) (Supplementary Figure 4). Thus, *Ptf1a* cKO mice showed decreased slow-wave activity production during the sleep/wake cycle; however, there were no significant changes in either wakefulness or NREMS in total amounts or duration.

**Figure 3:**
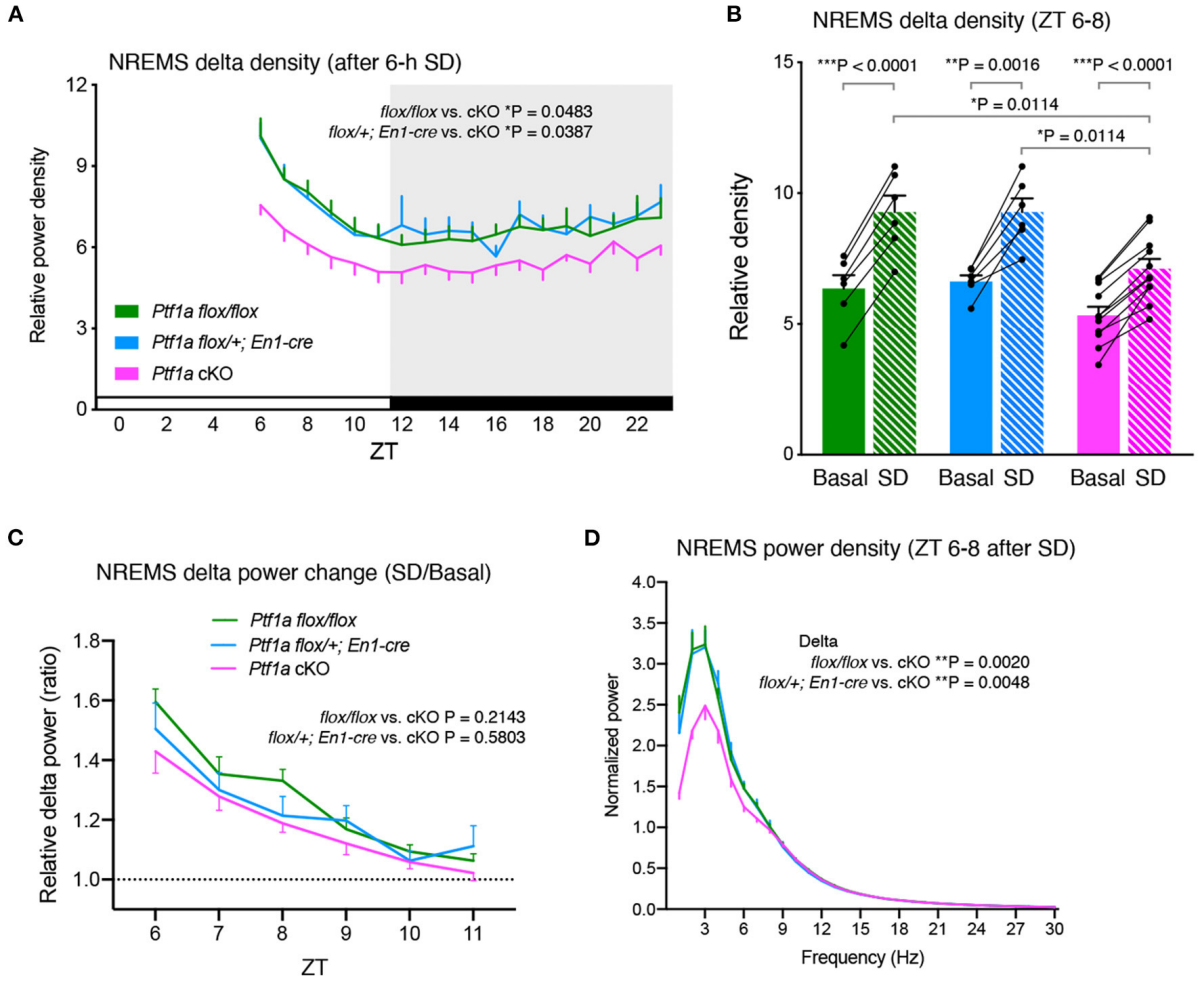
Sleep/wakefulness of adult male *Ptf1a* cKO mice after 6 h of sleep deprivation (SD). **(A)** Hourly NREMS delta power density of *Ptf1a*^*flox*/*flox*^ (*n* = 7), *En1*^*cre*/+^*; Ptf1a*^*flox*/+^ (*n* = 6), and *En1*^*cre*/+^*; Ptf1a*^*flox*/*flox*^ (*n* = 11) after 6 h of sleep deprivation. Mixed-effects analysis followed by Tukey's test. **(B)** NREMS delta density during ZT6–ZT8 of *Ptf1a*^*flox*/*flox*^ (*n* = 6), *En1*^*cre*/+^*; Ptf1a*^*flox*/+^ (*n* = 6), and *En1*^*cre*/+^*; Ptf1a*^*flox*/*flox*^ (*n* = 11) in the basal recordings and after 6 h of sleep deprivation. Two-tailed paired *t*-test for Basal vs. SD in each genotype. One-way ANOVA followed by Tukey's test for comparisons between genotypes. **(C)** NREMS delta power changes after sleep deprivation relative to basal condition during ZT6–ZT11. Mixed-effects analysis followed by Tukey's test. **(D)** EEG power spectra during ZT6–ZT8 of *Ptf1a*^*flox*/*flox*^ (*n* = 7), *En1*^*cre*/+^*; Ptf1a*^*flox*/+^ (*n* = 6), and *En1*^*cre*/+^*; Ptf1a*^*flox*/*flox*^ (*n* = 11) in NREMS after sleep deprivation. Mixed-effects analysis followed by Tukey's test for the delta- and theta-ranges. Data are the mean ± S.E.M. *P < 0.05. **P < 0.01. ***P < 0.001.

Next, to examine the homeostatic regulation of sleep/wakefulness in *Ptf1a* cKO mice, we performed sleep deprivation for 6 h with gentle handling from ZT0. After sleep deprivation, *Ptf1a* cKO mice showed increased NREMS delta density but the density was lower than control groups during the light and dark phases (Figure 3A). *Ptf1a* cKO mice had a lower NREMS time variation than *Ptf1a*^*flox*/*flox*^ mice (Supplementary Figure 3A). The changes in NREMS delta power in the first 2 h of recovery sleep (ZT6-8) were higher than the basal condition in all the genotypic groups (Figure 3B), whereas there were no significant changes in NREMS time in the first 2 h after sleep deprivation (Supplementary Figure 3B). Sleep deprivation enhanced NREMS delta power to a similar extent among the genotypic groups (Figure 3C). Similar to basal sleep/wakefulness, *Ptf1a* cKO mice had a lower delta power during NREMS than the two control groups for 2 h after sleep deprivation (Figure 3D), and this difference lasted at least 24 h after sleep deprivation. These results indicated that *Ptf1a* cKO mice exhibited enhanced delta power during NREMS after sleep deprivation; however, the degree of the enhanced delta power after sleep loss was similar to the control groups, suggesting normal homeostatic NREM sleep regulation in *Ptf1a* cKO mice.

### Mice Lacking Cerebellar Cortex Exhibited Normal NREMS Spindle Generation

**Figure 4:**
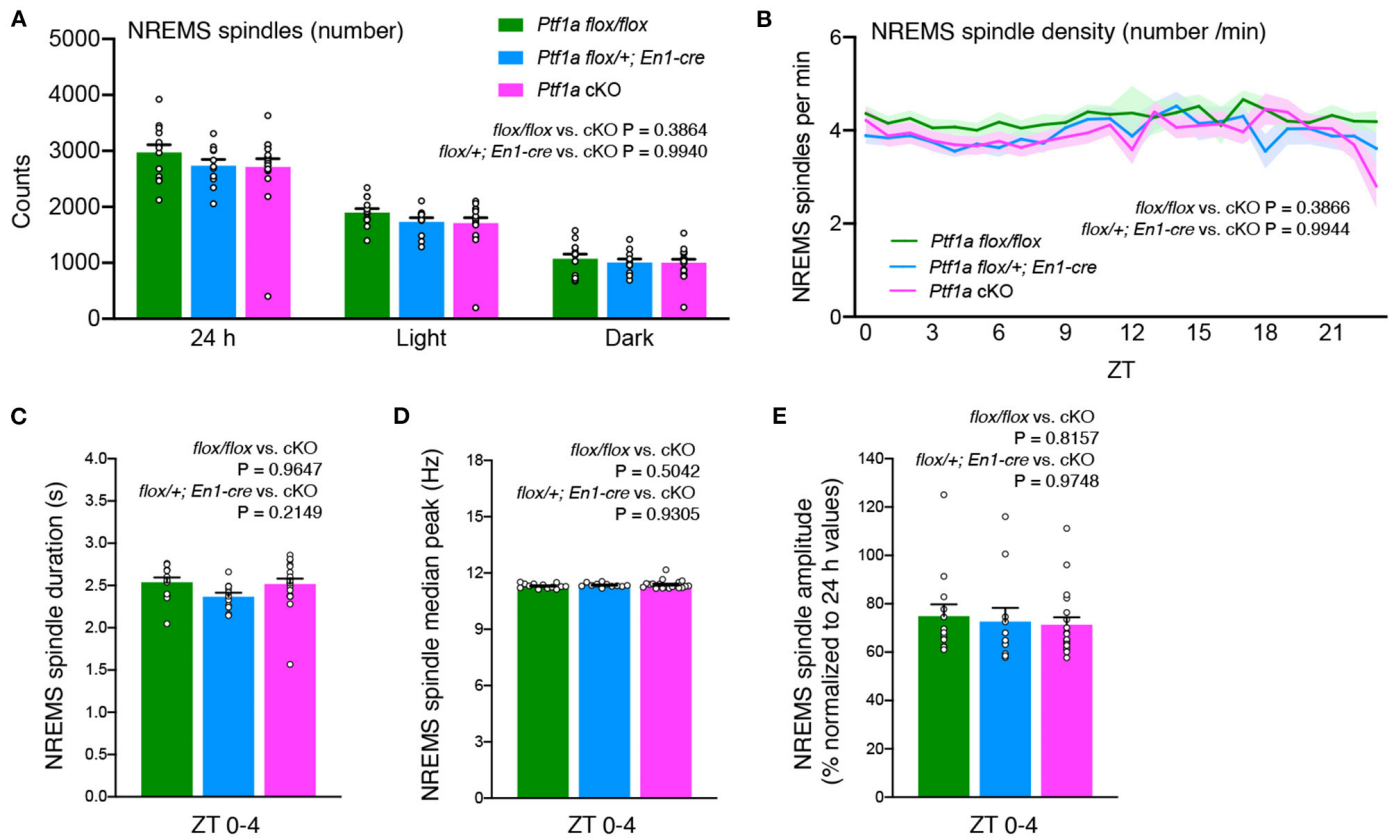
No significant differences are observed in NREMS spindles of adult male *Ptf1a* cKO mice. **(A)** NREMS spindle number of *Ptf1a* cKO and control mice over 24 h and during the light and dark phases under the basal condition. **(B)** Hourly NREMS spindle density (spindles/minute) over 24 h in *Ptf1a* cKO and control mice. **(C–E)** Quantitative analyses of NREMS spindle duration **(C)**, median peak frequency **(D)**, and normalized amplitude **(E)** during ZT0–ZT4 in *Ptf1a* cKO and control mice. *n* = 13 for *Ptf1a*^*flox*/*flox*^, *n* = 11 for *En1*^*cre*/+^*; Ptf1a*^*flox*/+^, *n* = 19 for *En1*^*cre*/+^*; Ptf1a*^*flox*/*flox*^. Mixed-effects analysis followed by Tukey's test. Data are the mean ± S.E.M.

The cerebellum is known to modulate thalamic activity through direct cerebello-thalamic projections (Gallay et al., 2008). Recently, a cerebellar contribution to neocortical sleep spindles *via* cerebello-thalamo-neocortical pathways was suggested in primates (Xu et al., 2021). *Ptf1a* cKO mice showed a similar NREMS spindle number during the light and dark phases (Figure 4A). There was no significant change in NREMS spindle density during the light and dark periods among the genotypic groups (Figure 4B). No significant differences in NREMS spindle duration, the median of the peak frequency of NREMS spindles, or the NREMS spindle amplitude were observed between genotypes (Figures 4C–E). These data indicate that the lack of the cerebellar cortex and related structures does not critically affect NREM sleep spindle generation.

### *Ptf1a* cKO Mice Showed No Impairment in Context and Cued Fear-Conditioning Behaviors

**Figure 5:**
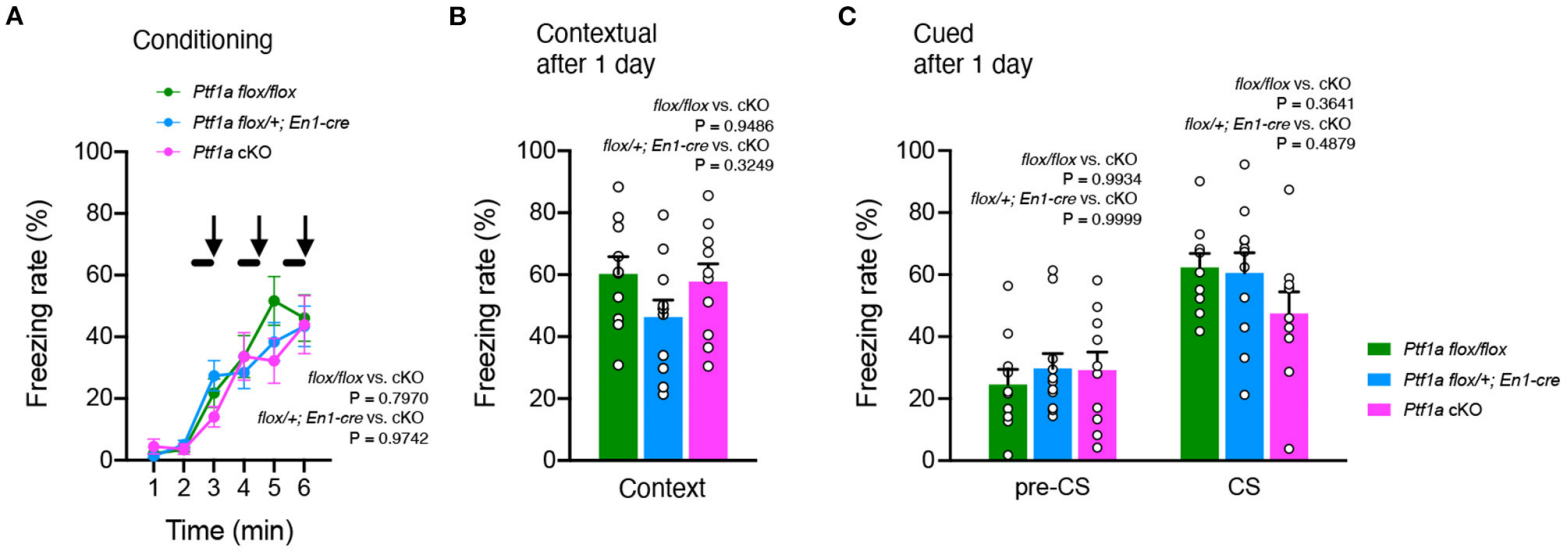
No significant differences were observed in fear-conditioning behavior of adult male *Ptf1a* cKO mice. **(A)** A percentage of time showing freezing during the training phase of *Ptf1a* cKO and control mice. A tone was presented for 30 s (black bars) followed by a 2 s foot shock (black arrows). Mixed-effects analysis. **(B,C)** Freezing rate of *Ptf1a* cKO and control mice during the contextual test **(B)** and the tone-cued test with altered context **(C)** conducted at 1 day after conditioning. *n* = 10 for *Ptf1a*^*flox*/*flox*^, *n* = 11 for *En1*^*cre*/+^*; Ptf1a*^*flox*/+^, *n* = 10 for *En1*^*cre*/+^*; Ptf1a*^*flox*/*flox*^. Mixed-effects analysis. Data are the mean ± S.E.M.

Finally, we performed a fear conditioning test, which consisted of three CS-US presentations, followed by a replacement (context) and a recall test of three acoustic CS presentations (cued) 1 day after to evaluate the short-term maintenance of fear memory. *Ptf1a*^*flox*/*flox*^ and *En1*^*Cre*/+^*; Ptf1a*^*flox*/+^ control mice had similar freezing levels in these tests. Shock stimulation during CS-US association was sufficient to induce freezing behavior at the same level in the control and *Ptf1a* cKO groups (Figure 5A). There was no significant difference in contextual fear between control and *Ptf1a* cKO mice (Figure 5B). When exposed to cued stimuli, there was no significant difference between groups (Figure 5C). These results suggest that genetic ablation of the cerebellar cortex does not affect short-term fear conditioning behaviors.

## Discussion

The present study showed that *Ptf1a* cKO mice exhibited lower EEG delta range power during wakefulness, NREMS and REMS. *Ptf1a* cKO mice have normal wake and NREMS time, decreased REMS time, and normal homeostatic responses to sleep deprivation.

The brain changes its activity across wakefulness, NREMS and REMS and generates characteristic neural oscillations such as slow waves and spindles (Adamantidis et al., 2019). Accordingly, the activity of cerebellar neurons changes through sleep-wake cycles (Canto et al., 2017). For instance, the firing rate of Purkinje cells in mice and cats was higher during wakefulness than during NREMS (Hobson and McCarley, 1972; Zhang et al., 2020), and that of fastigial nucleus neurons in cats tended to be higher during REMS than in other states (Palmer, 1979). Slow wave activity (<4 Hz) in EEG is prominent during NREMS and generated from the interaction between the cerebral cortex and thalamus (Adamantidis et al., 2019). In addition, the claustrum modulates slow waves by synchronizing the initiation of the down-state of cortical neurons (Narikiyo et al., 2020). Slow wave activity is also observed in the cerebellum as local field potential oscillations during natural sleep and anesthesia (Ros et al., 2009; Rowland et al., 2010; Xu et al., 2021; Torres-Herraez et al., 2022). It is generally believed that changes in cerebellar neuron activity during the sleep-wake cycle passively reflect activity of the brain as a whole because disruption of neocortical slow wave activity resulted in the loss of cerebellar slow waves (Ros et al., 2009), and the main direction of information was from the cerebral cortex to the cerebellum (Rowland et al., 2010; Xu et al., 2021). However, the current study showing lower EEG slow wave activity in *Ptf1a* cKO mice suggests that the cerebellar system can contribute to maintaining or enhancing neocortical slow wave activity. Consistently, EEG slow wave activity is associated with increased BOLD signals in the human cerebellum (Betta et al., 2021). Thus, the neural circuitry connecting the cerebral cortex, thalamus, and cerebellum, including direct cerebello-thalamic connections (Gallay et al., 2008), may coordinately work to generate EEG slow waves.

Spindles are characterized by waxing-waning 10–16 Hz oscillations generated in thalamic reticular nucleus circuits and thalamo-cortical projection neurons (Adamantidis et al., 2019). However, lesions of the dentate nucleus in cats increase sleep spindles (Shouse and Sterman, 1979). Directed coherence analysis of sleep spindles of freely moving primates exhibited the cerebellum to the neocortex direction (Xu et al., 2021). These findings indicate the possibility that the cerebellum modulates sleep spindles. However, *Ptf1a* cKO mice showed a frequency and duration of sleep spindles similar to control mice. Thus, although the cerebellar system may not contribute to the generation of sleep spindles, it remains possible that it regulates other functional parameters of sleep spindle, such as the phase of spindles relative to other oscillations.

Lesioning of the cerebellum in cats did not affect sleep (Jouvet, 1962; Hobson, 1965) or increased drowsiness and REMS and decreased wake and NREMS (Cunchillos and De Andrés, 1982). Lesion of the fastigial nucleus in cats increased total wake amount, decreased NREMS amount, and did not change REMS amount (Giannazzo et al., 1968). Moreover, lesions in the cerebellar vermis and hemispheres in cats showed a longer duration of NREMS and total REMS (de Andrés et al., 2011). Although these lesioning approaches indicated various changes in the sleep-wake cycle, *Ptf1a* cKO mice have normal wake and NREM sleep time. This seemingly contradictory result regarding wake amount may be due to compensation for the loss of the cerebellar system from the developmental stage.

*Ptf1a* cKO mice showed reduced REMS amount and episode duration compared to *En1*^*Cre*/+^*; Ptf1a*^*flox*/+^; and *Ptf1a*^*flox*/*flox*^ mice, and the number of REMS episodes was unchanged, which indicates the involvement of the cerebellar system in the maintenance of REMS, rather than the initiation of REMS. Another possibility is that r1-derived *Ptf1a*-lineage cells, which localize outside the cerebellar system, regulate REMS. A subset of glutamatergic *neurotensin(Nts)*-expressing *Atoh1*-lineage neurons in the dorsal pons-medulla region, which originates from the cerebellar rhombic lip, regulate the REMS amount (Hayashi et al., 2015; Kashiwagi et al., 2020). *Atoh1* and *Ptf1a* are expressed adjacent to each other in the cerebellar primordium (Hashimoto and Hibi, 2012; Hori and Hoshino, 2012), and approximately a half of *Nts* neurons are not in the *Atoh1*-lineage (Kashiwagi et al., 2020). Perhaps, there is a possibility that some r1-derived *Nts* neurons may arise from the nearby *Ptf1a*-expressing ventricular zone. In contrast, *Ptf1a*-lineage cells are not localized in the PPT and LDTg that contain cholinergic neurons which promote REMS (Scammell et al., 2017). In this study, we did not examine *Nts*-expressing neurons or cholinergic neurons in the mutant mice. Therefore, further studies on *Ptf1a*-lineage cells in the dorsal pons-medulla and the genetic background of the neuronal groups that regulate REMS are needed.

Inactivation of the cerebellar vermis or interpositus nucleus impaired fear conditioning behavior (Sacchetti et al., 2002), and long-term potentiation in the cerebellar cortex was involved in fear memory consolidation (Sacchetti et al., 2004). By chemogenetic and optogenetic manipulation, glutamatergic projections from the fastigial nucleus to the ventrolateral periaqueductal gray were implicated to controlling fear memory (Frontera et al., 2020). However, *Ptf1a* cKO mice formed amygdala-dependent and hippocampus-dependent fear memory, similar to control mice. However, slow wave activity during NREMS after learning plays an important role in memory consolidation (González-Rueda et al., 2018; Todorova and Zugaro, 2019).

The lower body weight in *Ptf1a* cKO mice may be due to their uncoordinated movements and unstable posture, which require extra energy expenditure to move around in the home cage, compared to wild-type mice. In addition, the deep cerebellar nucleus has excitatory monosynaptic inputs to the ventral tegmental area and modulates reward behavior (Carta et al., 2019). The anterior deep cerebellar nucleus regulates feeding behavior (Low et al., 2021). *Ptf1a* cKO mice may have abnormal rewarding behavior and food intake, although food intake was not examined in this study.

There are several limitations to this study. Although male mice were used in this study to reduce the number of mice, female *Ptf1a* cKO mice may yield different results, as we have showed that the sleep/wake behavior of female mice differs from that of male mice (Choi et al., 2021). Compensation for the loss of the cerebellum from the embryonic stage may have weakened the sleep phenotype of *Ptf1a* cKO mice. Therefore, the role of the cerebellar system in the regulation of sleep may be greater than suggested in the current study. Another limitation is that we cannot rule out the possibility that neurons outside the cerebellar system, including the hypothalamus and brainstem, are responsible for the sleep abnormalities in *Ptf1a* cKO mice. There are a small number of *Ptf1a*-lineage cells in the pons which are derived from the rhombomere 1 (Yamada et al., 2007). In addition, REMS-regulating neurons with direct fiber connections to the cerebellum, such as cholinergic neurons in the PPT and LDTg (Jaarsma et al., 1997), may be impaired by the loss of the cerebellum.

In summary, EEG/EMG analysis of *Ptf1a* cKO mice lacking the cerebellar system showed overall normal sleep/wake behavior except decreased REMS and a decrease in NREMS delta power.

## Data Availability Statement

The original data set used in this article will be made available by the authors, without undue reservation.

## Ethics Statement

The animal study was reviewed and approved by the Institutional Animal Care and Use Committee of the University of Tsukuba.

## Author Contributions

TF, MY, and HF: conceptualization and writing. TF, HT, KM, FA, NH-H, YI, SK, PS-C, HM, and MH: methodology. MH: resource. TF, HT, and HF: formal analysis. MY and HF: supervision. All authors contributed to the article and approved the submitted version.

## Funding

This work was supported by the World Premier International Research Center Initiative from MEXT to MY, AMED (JP21zf0127005 to MY), JSPS KAKENHI (17H06095 to MY and HF; 22H04918, 17H04023, 17H05583, 20H00567, to HF; 18KK0442 to HM; 24800088, 19H03142, 20H03416 to TF), Intramural Research Grant from NCNP to HM and MH, Funding Program for World-Leading Innovative R&D on Science and Technology (FIRST Program) from JSPS to MY, and Research Grant from Uehara Memorial Foundation, Naito Foundation, Astellas Foundation for Research on Metabolic Disorders to HF.

## Conflict of Interest

The authors declare that the research was conducted in the absence of any commercial or financial relationships that could be construed as a potential conflict of interest.

## Publisher's Note

All claims expressed in this article are solely those of the authors and do not necessarily represent those of their affiliated organizations, or those of the publisher, the editors and the reviewers. Any product that may be evaluated in this article, or claim that may be made by its manufacturer, is not guaranteed or endorsed by the publisher.
