# Supplementary material for: Mice Lacking Cerebellar Cortex and Related Structures Show a Decrease in Slow-Wave Activity With Normal Non-REM Sleep Amount and Sleep Homeostasis
Source: Front Behav Neurosci. 2022 Jun 2;16:910461. doi: 10.3389/fnbeh.2022.910461 (PMC9203121; doi:10.3389/fnbeh.2022.910461)
Supplement: Supplementary file 1 [file Data_Sheet_1.docx]

Supplementary Material

# Supplementary Data

## Supplementary Figures

**
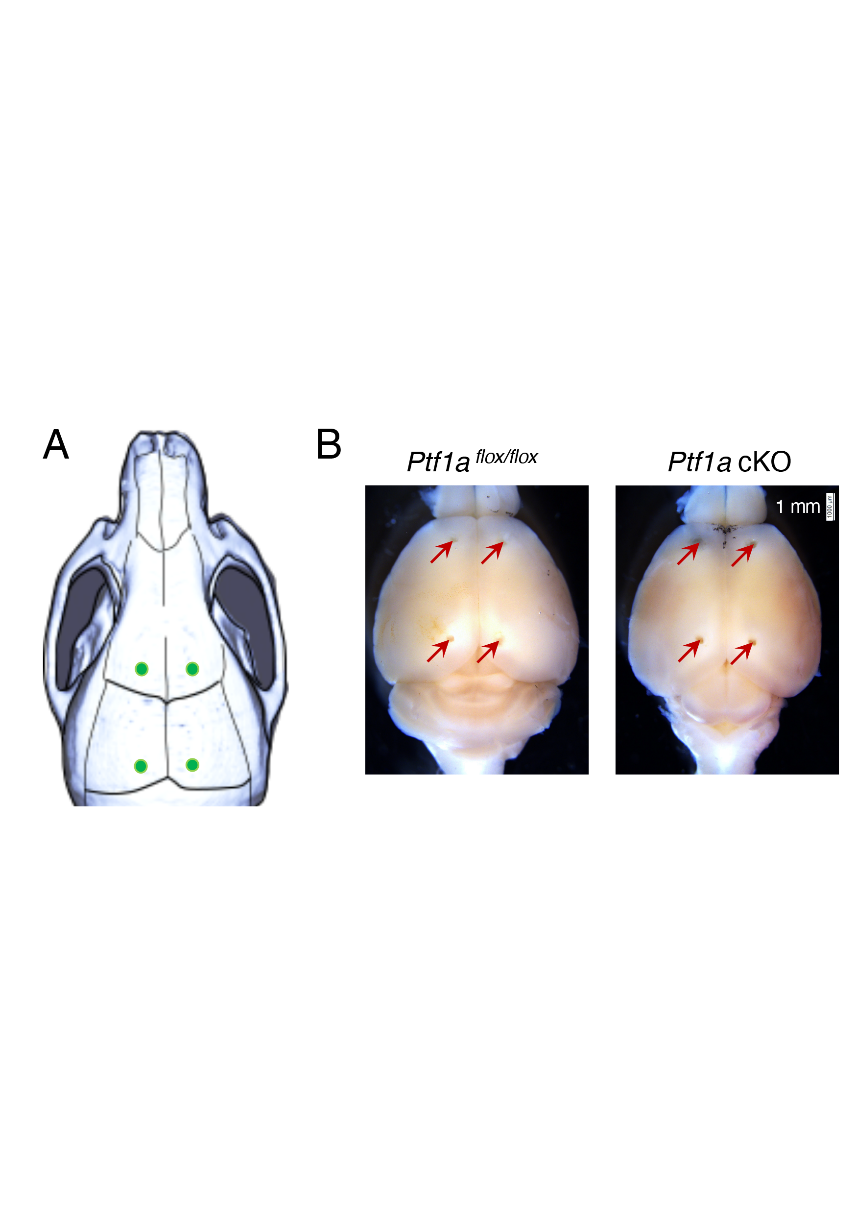
**

**Supplementary Figure 1.** **Positions of four EEG electrode pins.**

(**A**) Representative image of adult mouse skull. Green dots indicate four hole positions for insertion of EEG electrode pins. (**B**) Whole brains of adult male control (left: *Ptf1a^flox/flox^*) and *Ptf1a* cKO (right: *En1^Cre/+^; Ptf1a^flox/flox^*) mice after EEG/EMG recording are shown from dorsal view. Red arrows indicate each point of EEG electrodes on the mouse cortex. Gross structural differences of the cortical surface and positions of EEG pins in *Ptf1a* cKO mice were not observed. Scale bar, 1 mm.


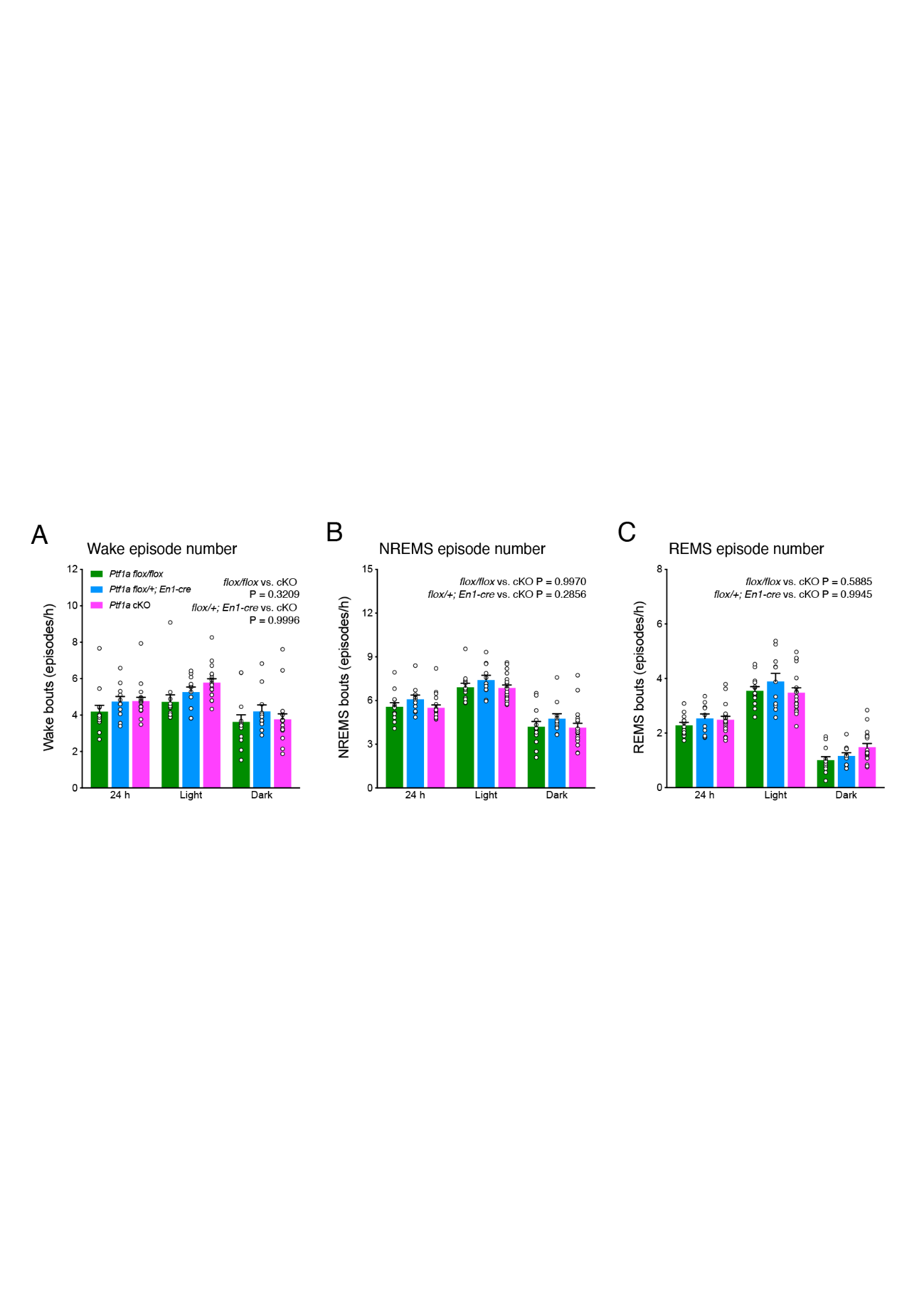


**Supplementary Figure 2.** **Sleep/wake behavior of adult male *Ptf1a* cKO mice: episode number.**

(**A-C**) Episode bouts number of wake (**A**), NREMS (**B**), and REMS (**C**) of *Ptf1a* cKO and control groups. Two-way ANOVA with Sidak's multiple comparisons test. n = 13 for *Ptf1a^flox/flox^*, n = 11 for *En1^cre/+^; Ptf1a^flox/+^*, n = 19 for *En1^cre/+^; Ptf1a^flox/flox^*. Data are mean ± S.E.M.


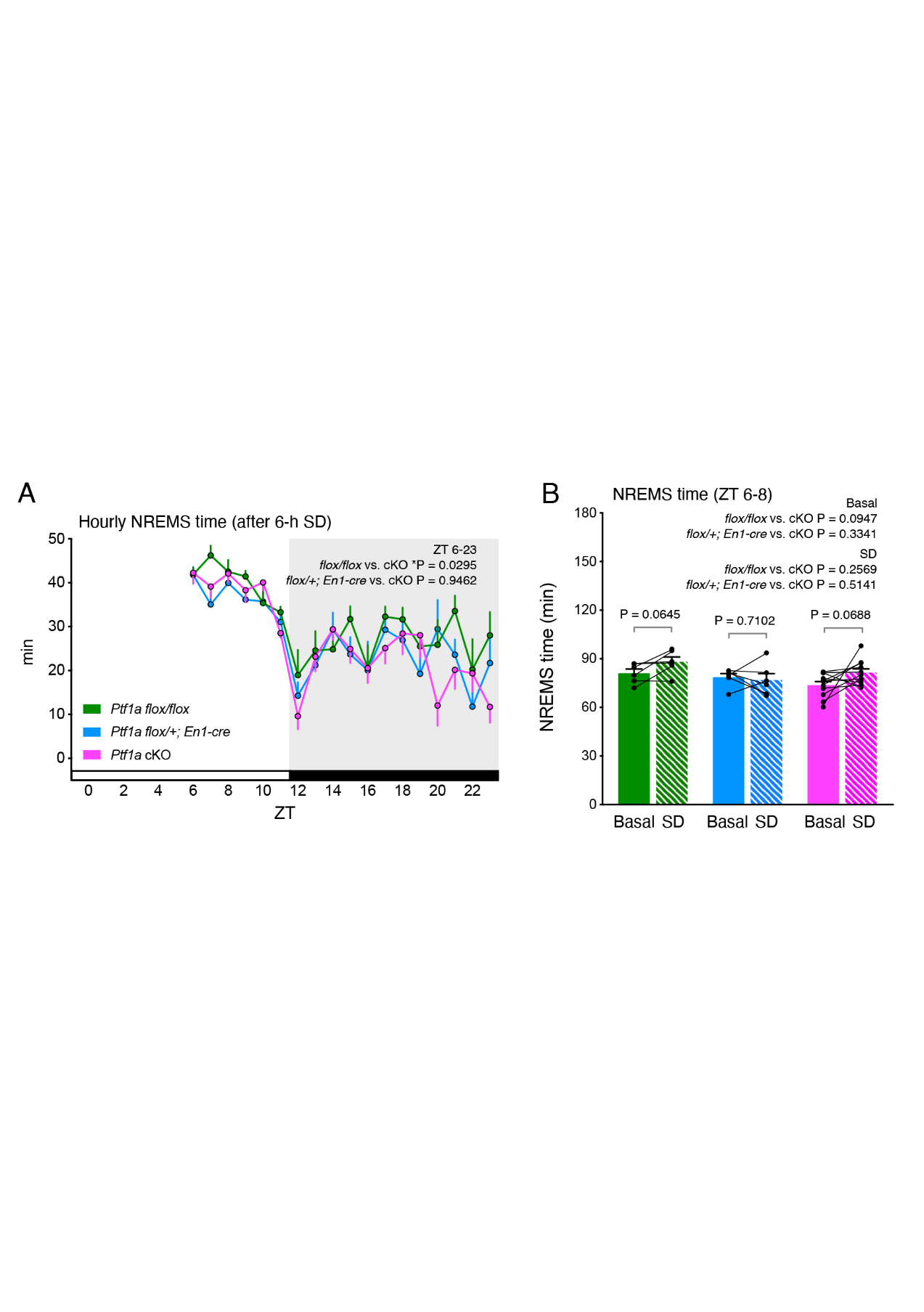


**Supplementary Figure 3.** **NREM sleep of adult male *Ptf1a* cKO mice after 6 h of sleep deprivation.**

(**A**) Daily variations of NREMS of *Ptf1a^flox/flox^* (n = 7), *En1^cre/+^; Ptf1a^flox/+^* (n = 6), and *En1^cre/+^; Ptf1a^flox/flox^* (n = 11) after 6-h sleep deprivation. Mixed-effects analysis followed by Tukey's test. Data are mean ± S.E.M. *P < 0.05. (**B**) NREMS time during ZT6 to ZT8 of *Ptf1a^flox/flox^* (n = 6), *En1^cre/+^; Ptf1a^flox/+^* (n = 6), and *En1^cre/+^; Ptf1a^flox/flox^* (n = 11) in the basal recordings and after 6-h sleep deprivation. Two-tailed paired t test for Basal vs SD in each genotype. One-way ANOVA followed by Tukey's test for comparisons between genotypes. Data are mean ± S.E.M.


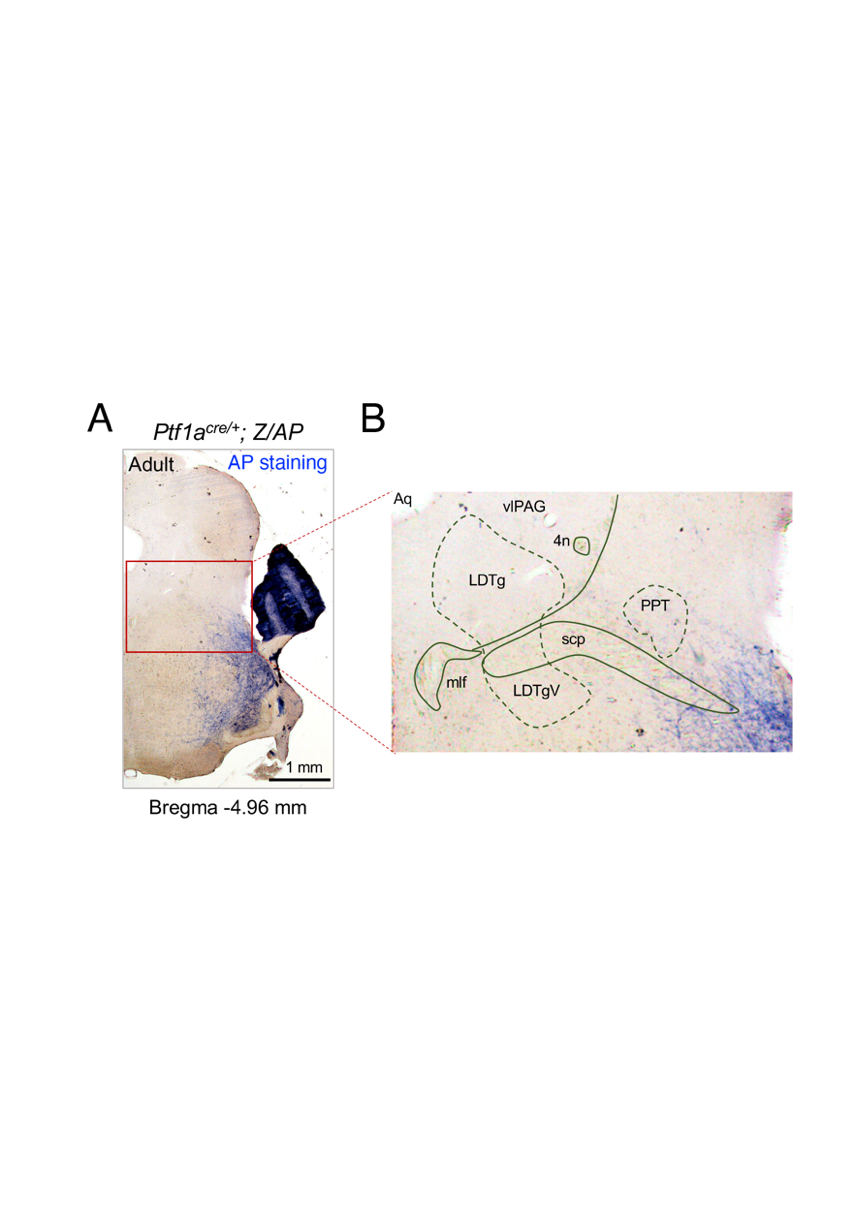


**Supplementary Figure 4.** **Hindbrain** ***Ptf1a*-lineage cells are not located in the laterodorsal tegmental nucleus and pedunculopontine tegmental nucleus.**

(**A**) Coronal section from adult *Ptf1a^cre^* heterozygous mouse with *Z/AP* reporter stained with NBT-BCIP by alkaline phosphatase. Half side of brain is represented. Scale bar, 1 mm. (**B**) Magnified view of red boxed region including LDTg and PPT in panel A is shown. In dorsal pons region, some of projections from *Ptf1a*-lineage cells are observed, although there are no signals of cell bodies of *Ptf1a*-lineage in LDTg and PPT. Aq: aqueduct, vlPAG: ventrolateral periaqueductal gray, 4n: fourth cranial nerve, LDTg: laterodorsal tegmental nucleus, LDTgV: ventral part of LDTg, PPT: pedunculopontine tegmental nucleus, mlf: medial longitudinal fasciculus, scp: superior cerebellar peduncle. Neuroanatomical nomenclatures and abbreviations are based on Flanklin and Paxinos (K.B.J. Flanklin and G. Paxinos. The Mouse Brain in Stereotaxic Coordinates, 3rd edition, Academic Press 2008).

## Supplementary Movies


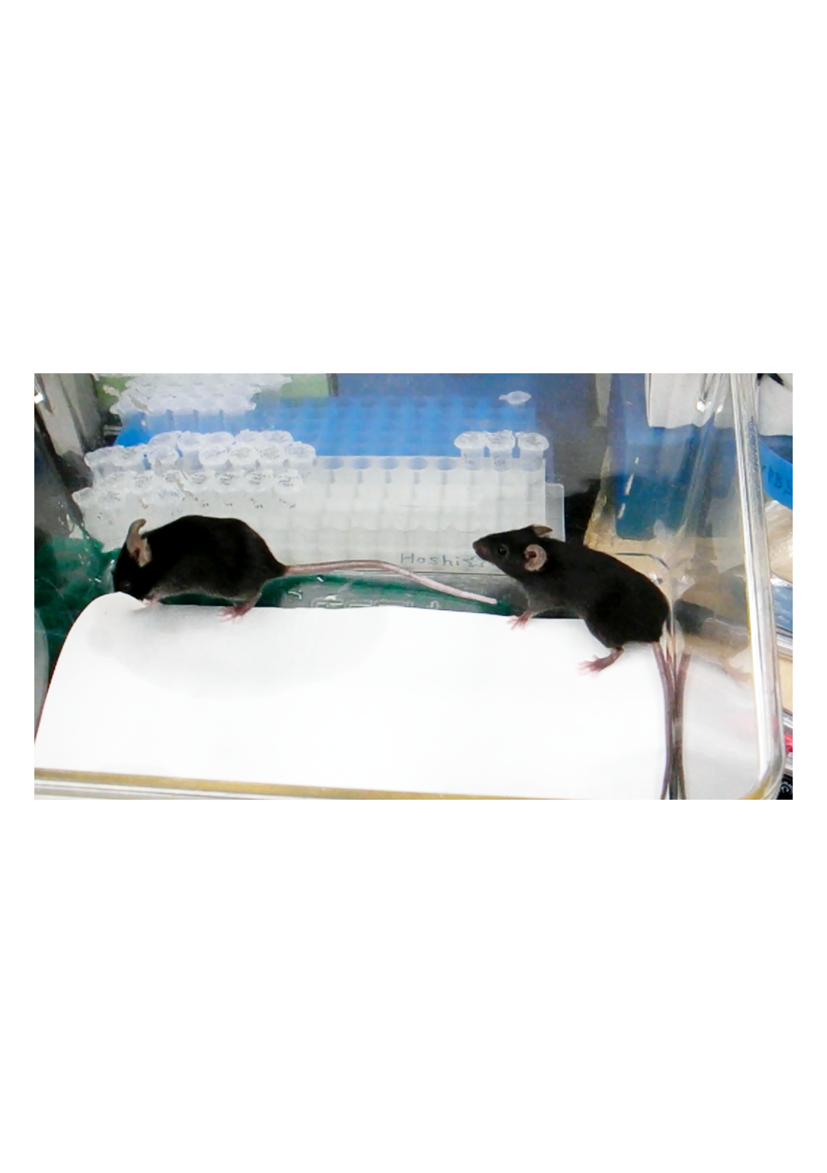


**Supplementary Movie 1.** **Appearance and movement of adult *Ptf1a* cKO and control mice.**

Left: control mouse. Right: *En1^cre/+^; Ptf1a^flox/flox^* mouse. Please see a movie from the link below.

(<https://www.frontiersin.org/articles/file/downloadfile/910461_supplementary-materials_videos_1_mp4/octet-stream/Video%201.MP4/1/910461>)
